# Multimodal physiological signal emotion recognition based on multi-head cross attention with representation learning

**DOI:** 10.3389/fpsyt.2025.1713559

**Published:** 2025-12-11

**Authors:** Shihang Ding, Lin Ma, Haifeng Li

**Affiliations:** Faculty of Computing, Harbin Institute of Technology, Harbin, China

**Keywords:** emotion recognition, multimodal, cross attention, feature fusion, physiological signal

## Abstract

**Introduction:**

Physiological signals offer a significant advantage in the field of emotion recognition due to their objective nature, as they are less susceptible to volitional control and thus provide a more veridical reflection of an individual's true affective state. The use of multimodal physiological signals enables a more holistic characterization of emotions, establishing multimodal emotion recognition as a critical area of research. However, existing multimodal fusion methods often fail to capture the complex, dynamic interactions and correlations between different modalities. Consequently, they exhibit limitations in fully leveraging complementary information from other physiological signals during the feature learning process.

**Methods:**

To address these shortcomings, we propose a novel framework for multimodal physiological emotion recognition. This framework is designed to comprehensively learn and extract features from multiple modalities simultaneously, effectively simulating the integrative process of human emotion perception. It utilizes a dual-branch representation learning architecture to process electroencephalography (EEG) and peripheral signals separately, providing high-quality inputs for subsequent feature fusion. Furthermore, we employ a cross attention mechanism tailored for multimodal signals to fully exploit the richness and complementarity of the information. This approach not only improves the accuracy of emotion recognition but also enhances robustness against issues such as missing modalities and noise, thereby achieving precise classification of emotions from multimodal signals.

**Results:**

Experimental results on the public DEAP and SEED-IV multimodal physiological signal datasets confirm that our proposed model demonstrates superior performance in the emotion classification task compared to other state-of-the-art models. Our findings prove that the proposed model can effectively extract and fuse features from multimodal physiological signals.

**Discussion:**

These results underscore the potential of our model in the domain of affective computing and hold significant implications for research in healthcare and human-computer interaction.

## 1 Introduction

Emotion, a cerebral response to specific stimuli, constitutes a crucial component of human intelligence (1). The endeavor to integrate emotion as a key factor in Human-Computer Interaction (HCI) and to endow machines with the capacity to perceive and understand human emotions has rapidly evolved into a burgeoning interdisciplinary research field known as Affective Computing (2). Affective Computing operates at the intersection of cognitive science and computer science, with the goal of enabling computers to recognize, interpret, and even express emotions, thereby developing artificial intelligence capable of emotional perception, comprehension, and regulation (3). With the advancement of AI, emotion recognition technologies have progressed significantly, paving the way for more effective and intuitive human-computer communication. As a primary research direction within Affective Computing, emotion recognition has found extensive applications across diverse domains, including human-computer interaction, education and teaching, and medical rehabilitation (4–6).

Traditional methods for emotion recognition often rely on overt cues such as facial expressions, voice, and text (7). In contrast, physiological signals are less susceptible to conscious control and influence, and they reflect an individual’s true emotional state, thus affording them greater reliability and robustness in the field of emotion recognition (8). As emotional responses and changes are intrinsically linked to the nervous system, they induce reactions in various physiological signals, including electroencephalography (EEG), electromyography (EMG), galvanic skin response (GSR), and electrocardiography (ECG) (9–12). Among these, EEG has become a primary focus for physiological signal-based emotion recognition due to its advantages, such as being non-invasive and having a high temporal resolution, which allows for better capture of the dynamic changes in brain activity during affective processing (13). Furthermore, since eye-tracking data reflects the brain’s visual attention and cognitive load—both closely related to emotion processing—the fusion of EEG with other concurrently recorded modalities like eye-tracking and GSR is attracting increasing attention from researchers (14). Despite progress, emotion recognition based on a single modality still faces significant bottlenecks, as unimodal signals suffer from insufficient informational richness, signal noise, and individual differences, limiting the broader application of these techniques (15). Under the combined influence of factors such as time, subject variability, and emotional state, emotion regulation mechanisms lead to diverse physiological responses, corresponding to complex multimodal physiological signals (16). Therefore, fusing multiple physiological modalities is widely regarded as a promising solution for building robust and stable emotion recognition systems (17). By integrating EEG with other physiological signals, a more comprehensive description and characterization of emotional states can be achieved (18).

Emotion recognition based on multimodal physiological signals primarily investigates the process of analyzing an individual’s affective state through the synthesis of multiple physiological signals. This process involves collecting and processing these signals and applying machine learning or deep learning techniques to accurately identify and classify human emotions (19). Yin et al. (20) proposed an ensemble classifier based on a multilayer-fused stacked autoencoder (MESAE) to recognize emotions, wherein hidden layer neurons extract high-level features from each modality, achieving good recognition performance. Tang et al. (21) extended the traditional autoencoder by proposing a bimodal deep denoising autoencoder that also considers temporal information for multimodal emotion recognition. Qiu et al. (22) introduced a multi-view emotion recognition framework using Deep Canonical Correlation Analysis (DCCA), which jointly learns the parameters of multi-view nonlinear transformations to maximize their correlation, finding that DCCA effectively learns highly correlated representations to improve classification accuracy. Zhu et al. (23) employed a Multi-Hypergraph Neural Network (MHGNN) to identify emotions from physiological signals, using a multi-hypergraph structure to represent inter-subject correlations and generating a hypergraph for each physiological signal type, which more accurately depicted the true biological response process. Wu et al. (24) proposed an emotion-related key subnet selection algorithm and used DCCA to pass network features along with eye-tracking features to a multimodal model, achieving accurate mining of inter-channel information. Cheng et al. (25) introduced a dense graph convolutional network based on a joint cross attention mechanism to integrate the spatial topology, consistency, and complementarity of multimodal data within a unified network framework, performing intra-modal and inter-modal cross attention fusion according to the characteristics of each modality. Their experimental results demonstrated that the model could effectively extract and fuse multimodal features.

While significant progress has been made in emotion recognition using multimodal physiological signals, several key shortcomings persist. The evolution of emotion is manifested not only in the dynamics of a single signal but, more critically, in the complex synergistic changes between brain states and peripheral signals. Simple fusion methods, such as feature concatenation or averaging, merely “stack” information together and are incapable of capturing the dynamic, non-linear interactions between different modalities. Furthermore, early fusion approaches, which concatenate raw signals before feeding them into a neural network, produce a single mixed feature sequence. This approach fails to capture the intricate, dynamic interactions and correlations between modalities and, critically, precludes the use of more sophisticated fusion mechanisms. The focus of other mainstream architectures (such as GNNs) is intra-modal. They explicitly and meticulously model the spatial dependencies between EEG electrodes by constructing graph structures. This is undoubtedly powerful in single-modality EEG analysis. In contrast, the focus of our framework is inter-modal. The core task of our designed cross-attention module is to dynamically capture the complex correlations between the EEG feature sequence and the peripheral signal feature sequence. A primary advantage of our architecture lies in its modular dual-branch design. It first respects the heterogeneity of the different modalities by learning the temporal dynamic features of each modality in separate, independent branches. The benefit of this design is that it provides two high-quality, information-pure, and decoupled high-order feature sequences for the subsequent cross-attention module. This stands in stark contrast to early fusion (which mixes raw signals) or GNNs (which focus on internal EEG topology). Consequently, how to effectively leverage information from other physiological modalities for feature learning and how to optimally fuse these signals for superior emotion recognition performance have become pressing challenges to be addressed. Therefore, a new fusion framework is needed—one that can independently model each modality and, on that basis, achieve deep inter-modal fusion in a more robust and flexible manner.

**Figure 1:**
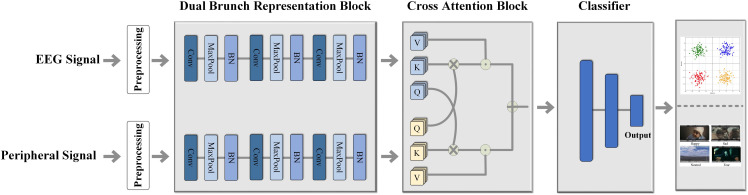
The overall framework of the proposed model.

To address the aforementioned problems in the field of multimodal emotion recognition, this paper proposes a method based on cross attention and representation learning. The overall framework of the proposed model is illustrated in **Figure 1**. The main contributions of this work are as follows: First, we propose a multimodal physiological emotion recognition framework that comprehensively learns and extracts information from multiple modalities, aiming to effectively model the processes of human emotion perception and recognition. Second, we design a dual-branch representation learning architecture to process electroencephalography (EEG) and peripheral signals separately, which provides ideal inputs for subsequent feature fusion and enhances the model’s interpretability through its modular design. Third, we design a multi-head cross attention mechanism tailored for multimodal signals to fully leverage the richness and complementarity of the information, improving emotion recognition accuracy while better handling issues such as modal-specific noise and missing data to achieve accurate classification. Finally, we conducted comprehensive and rigorous comparative experiments against several representative baseline models. The results demonstrate that our proposed method significantly improves the performance of emotion recognition from multimodal physiological signals, effectively validating the superiority of our model.

## 2 Materials and methods

### 2.1 Datasets

To evaluate the performance of our proposed model, we utilized two publicly available multimodal emotion datasets: DEAP (26) and SEED-IV (27). The specifics of these datasets are detailed below.

The Database for Emotion Analysis using Physiological Signals (DEAP) is a multimodal dataset collected for emotion research through cognitive experiments. In the experimental paradigm, music videos were used as stimuli to elicit emotional responses from participants. The dataset comprises physiological data from 32 participants (16 male, 16 female) across a total of 48 channels, including electroencephal ography (EEG), electrooculography (EOG), and galvanic skin response (GSR). The EEG signals were acquired at a sampling rate of 512 Hz using a 32-channel system arranged according to the international 10–20 standard. The remaining channels include 12 peripheral physiological signals, 3 unused channels, and 1 status channel. During the experiment, participants were asked to watch 40 one-minute-long music videos, each associated with a different emotional tone. After viewing each video, participants performed a self-assessment, rating their levels of valence, arousal, and other dimensions on a scale from 1 to 9. Each data trial is 63 seconds in duration, which includes a 3-second baseline recording prior to the formal experiment and 60 seconds of data collected during the video viewing.

**Figure 2:**
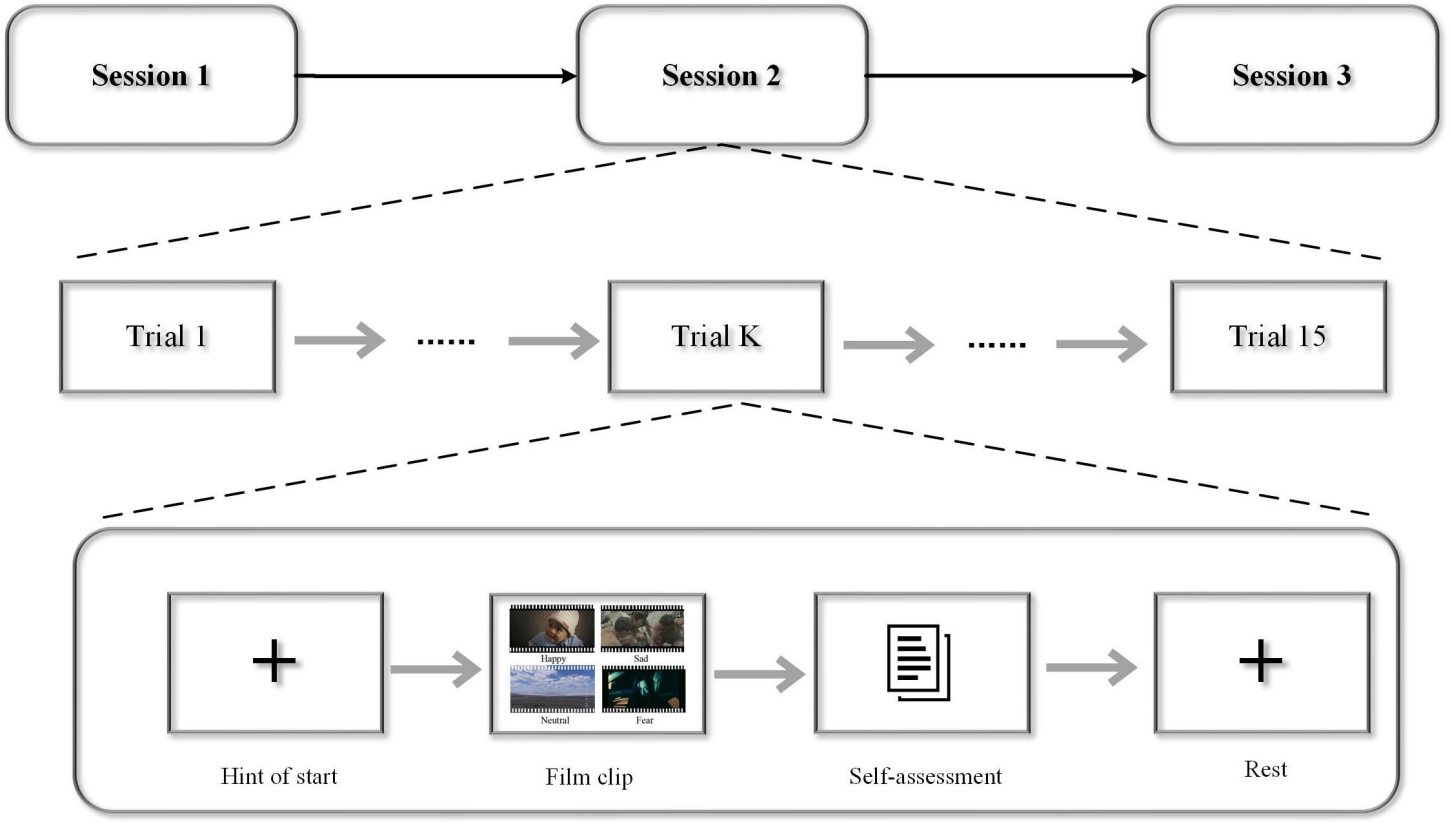
SEED-IV dataset emotion perception experimental paradigm.

**Figure 3:**
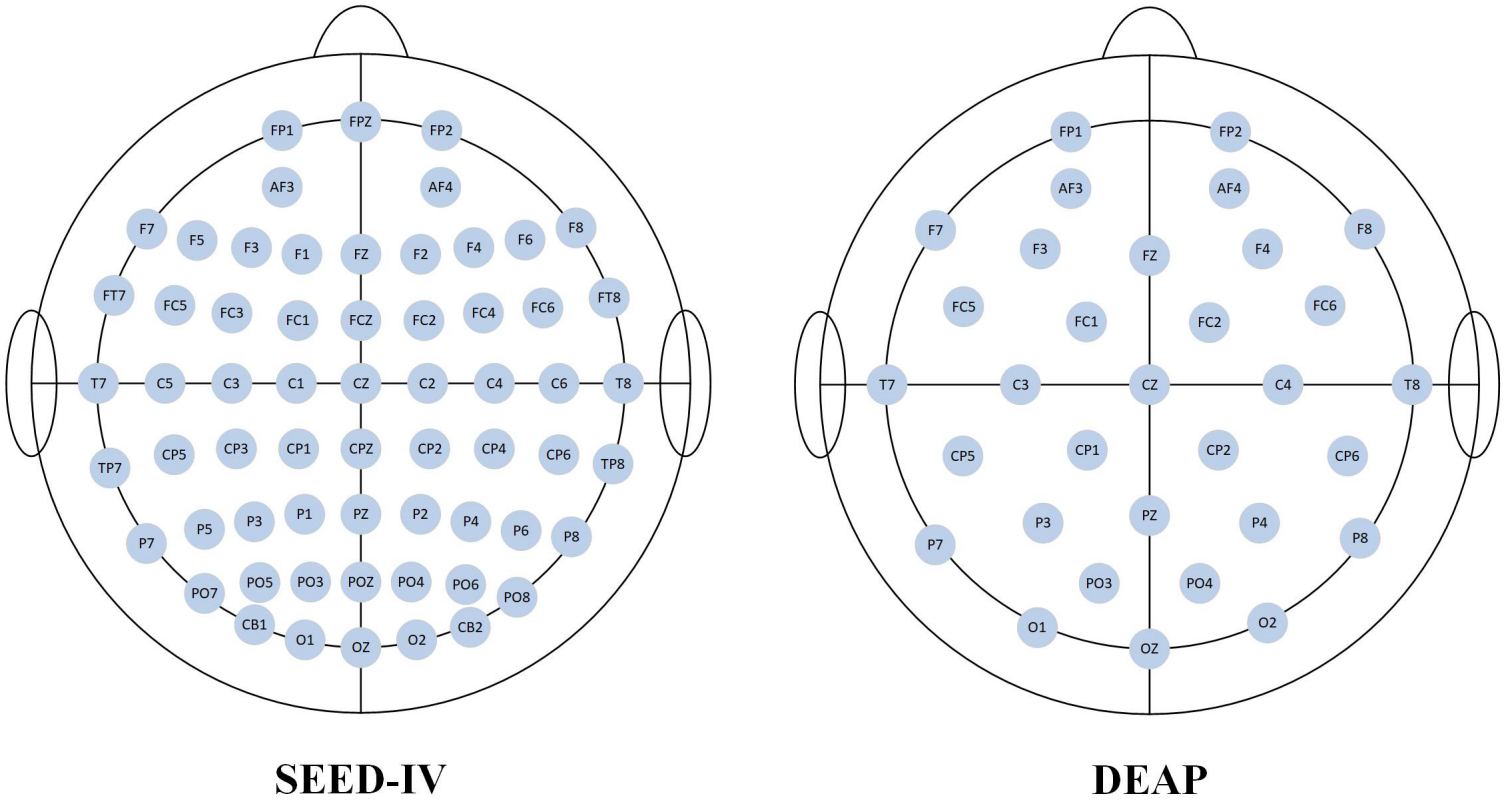
Distribution of electrode positions for two datasets.

The SJTU Emotion EEG Dataset IV (SEED-IV) is a multimodal dataset collected by Professor Bao-Liang Lu’s team at Shanghai Jiao Tong University, containing both EEG and eye-tracking data. The experiment employed emotional film clips to induce affective states in the subjects. Compared to other stimuli such as audio or music alone, film clips offer a significant advantage as they integrate both video and audio channels, providing a more immersive and realistic scenario for the participants, thereby eliciting stronger and more authentic emotional and psychological changes. To ensure clarity, each video clip was selected to induce a single, discrete emotional category. The stimuli for the dataset were chosen from 24 video clips of varying emotional content, each approximately 2 minutes in length. The dataset encompasses four distinct emotion classes: happy, neutral, sad, and fear. A total of 15 subjects (8 female, 7 male) participated in the experiment. EEG signals were continuously recorded at a sampling rate of 1000 Hz using a 64-channel Neuroscan system, with electrodes placed according to the standard 10–20 system. Eye-tracking data were collected using SMI-ETG eye-tracking glasses. **Figure 2** shows a schematic diagram of the experimental procedure for affective EEG data collection. **Figure 3** illustrates the electrode position distribution for the two datasets used in this study. The details of the DEAP and SEED-IV dataset are presented in **Table 1**.

**Table 1:** Details of the DEAP and SEED-IV dataset.

Database	DEAP	SEED-IV
Subjects	32	15
Stimulus	Musical videos	Movie clips
Trials	40	24
Sessions	1	3
Data modalities	7	2
Sampling rate	128	200
Emotional classes	2	4

### 2.2 Data preprocessing

For the DEAP dataset, we followed the same preprocessing procedure as described in Liu et al. (28). First, the initial 3-second baseline period was removed from the raw EEG data. The signals were then downsampled to a sampling rate of 128 Hz. Electrooculogram (EOG) artifacts were removed using the method detailed in the original DEAP publication (26). Subsequently, a band-pass filter between 4 Hz and 45 Hz was applied to eliminate low-frequency drift and high-frequency noise. Finally, the preprocessed EEG signals were decomposed into four distinct frequency bands using filtering: theta (*θ*, 4–8 Hz), alpha (*α*, 8–13 Hz), beta (*β*, 13–30 Hz), and gamma (*γ*, 30–45 Hz).

For the SEED-IV dataset, we adopted the preprocessing steps outlined in [25]. The raw EEG signals were first downsampled to 200 Hz. A band-pass filter from 1 Hz to 70 Hz was then applied to the data to isolate the desired frequency range and remove power-line interference. As EEG signals recorded during the experiment were contaminated by eye-movement artifacts, Independent Component Analysis (ICA) was employed to identify and remove these artifacts. Following this, the cleaned EEG signals were decomposed into five frequency bands via filtering: delta (*δ*, 1–4 Hz), theta (*θ*, 4–8 Hz), alpha (*α*, 8–14 Hz), beta (*β*, 14–31 Hz), and gamma (*γ*, 31–50 Hz).

### 2.3 Dual-branch representation learning module

For the task of multimodal emotion classification, we designed a dual-branch architecture to extract features and recognize emotions from electroencephalography (EEG) and peripheral signals in separate streams. The model architecture is illustrated in **Figure 1**. It should be noted that for the DEAP dataset, peripheral signals refers to physiological signals such as GSR and EOG, whereas for the SEED-IV dataset, it refers to eye-tracking signals. Both branches share an identical network architecture, which is designed to hierarchically extract dynamic features indicative of emotional states from the input time-series data. Each branch receives a preprocessed time-series segment as input. This input sequence first passes through a one-dimensional convolutional layer (Conv1D) to capture low-level, local temporal patterns from the raw features. For an input *X* and the *j*-th convolutional kernel *W_j_*, the corresponding output feature map *Y_j_* is calculated by Equation 1: where *σ* represents the activation function, and *W_j_* and *b_j_* are the learnable kernel weights and bias term of the layer, respectively.

(1) $$Y_{j}=\sigma (\text{Conv }(X,W_{j})+b_{j})$$

This is followed by a max-pooling layer (MaxPooling1D), which serves to increase the receptive field of subsequent layers while reducing computational complexity. To improve training efficiency and model stability, a Batch Normalization layer is incorporated. For a given activation value *x_i_* at the input to this layer, its normalized output, denoted as ${\hat{x}}_{i}$, is calculated by Equation 2:

(2) $${\hat{x}}_{i}=\gamma \frac{x_{i}-\mu_{B}}{\sqrt{\sigma_{B}^{2}+\in }}+\beta$$

Finally, a Dropout layer is applied to randomly deactivate a fraction of neurons, effectively preventing model overfitting. Through the cascaded processing of three such convolutional blocks, the original features are ultimately transformed into a high-level feature sequence. This output sequence is not only effectively downsampled in the temporal dimension but also encapsulates key dynamic patterns from the original features across different time scales. This feature sequence then serves as the input for the subsequent multi-head cross attention module for the deep fusion of multimodal information.

### 2.4 Multi-head attention mechanism

To facilitate the effective extraction of features from each signal modality, our model incorporates an attention mechanism, for which Scaled Dot-Product Attention serves as the core computational unit. The output of the attention function is mathematically defined by Equation 3:

(3) $$\text{Attention}(Q,K,V)=\text{softmax }(\frac{QK^{T}}{\sqrt{d_{k}}})V$$

where *Q* represents the Query matrix, *K* represents the Key matrix, and *V* represents the Value matrix and *d_k_* is the dimension of the *K*.

To enable the model to learn associations between different representation subspaces of the various modalities and to further enhance its expressive power, this study employs a multi-head attention mechanism. This involves performing *h*, independent linear projections of *Q*, *K*, *V*, and then feeding these projected versions into their respective Scaled Dot-Product Attention modules in parallel, enabling simultaneous attention to information from different representation subspaces. The specific process is as follows:

The input *Q*, *K*, *V* are each linearly transformed using *h* distinct sets of learnable weight matrices $\left(W_{i}^{Q},W_{i}^{K},W_{i}^{V}\right)$ to generate *h* sets of lower-dimensional queries, keys, and values. We obtain the following formula Equation 4.

(4) $${\text{head}}_{i}=\text{Attention }(QW_{i}^{Q},KW_{i}^{K},VW_{i}^{V})$$

where $W_{i}^{Q}\in {\mathbb{R}}^{d_{k}/h},W_{i}^{K}\in {\mathbb{R}}^{d_{k}/h},W_{i}^{V}\in {\mathbb{R}}^{d_{v}/h}$ The *h* attention heads then compute the Scaled Dot-Product Attention in parallel, generating *h* output matrices. Subsequently, the outputs of these heads are concatenated. Finally, the concatenated matrix is passed through a linear projection matrix *W* to map it back to the original model dimension, yielding the final output of the multi-head attention layer by Equation 5.

(5) $$\text{MultiHead}(Q,K,V)=\text{Concat }({\text{head}}_{1},\ldots ,{\text{head}}_{h})\;W$$

### 2.5 Multimodal cross attention module

To capture the complex, dynamic relationships between the two signal modalities, the representation of one modality is used as the Query to attend to relevant parts of the other modality, thereby enabling an effective fusion of the multimodal signals. Specifically, each new feature vector in an enhanced sequence is computed by taking a weighted sum of the features from the entire peripheral signal sequence. The weights are determined by the degree of relevance between the current EEG feature and all features in the peripheral sequence. The multimodal cross attention module thus establishes an accurate correspondence between patterns in the EEG signal and relevant information within the peripheral signals. Furthermore, to enable the model to learn associations from different perspectives and subspaces, a multi-head attention mechanism is used. The outputs of the individually computed attention heads are concatenated to further enhance the model’s expressive power.

Let $Attention_{EEG}$ represent the cross attention output derived from the electroencephalogram (EEG) signals, and let $Attention_{PERI}$ represent the cross attention output derived from the peripheral device signals. These two outputs are then fused to form a final feature representation as Equations 6, 7.

(6) $$Attention_{EEG}=MultiHead(Q_{PERI},K_{EEG},V_{EEG})$$

(7) $$Attention_{PERI}=MultiHead(Q_{EEG},K_{PERI},V_{PERI})$$

During model training, the resulting feature vector is passed through a fully connected layer for dimensionality reduction and is then used to generate prediction labels and the final classification outcome. To evaluate the classification results of our model, we use Accuracy as the performance metric, defined as follows by Equation 8:

(8) $$Accuracy=\frac{\left(TP+TN\right)}{\left(TP+TN+FP+FN\right)}$$

This formula is presented as an example for a binary classification task. The denominator represents the total number of samples, which is the sum of True Positives (TP), True Negatives (TN), False Positives (FP), and False Negatives (FN). The numerator is the sum of TP and TN, which corresponds to the total number of correctly predicted samples.

### 2.6 Feature engineering

For the DEAP dataset, we extracted Differential Entropy (DE) features from the four preprocessed EEG frequency bands. The DE features were computed using a Short-Time Fourier Transform (STFT) with a 4-second non-overlapping Hanning window. The other peripheral physiological signals were also downsampled to 128 Hz, had the initial 3-second baseline removed, and were segmented into 60-second trials. These peripheral data were consolidated into 8 channels, including 2 electrooculogram (EOG) channels, 2 electromyogram (EMG) channels, 1 galvanic skin response (GSR) channel, 1 skin temperature (SKT) channel, 1 respiration (RSP) channel, and 1 blood volume pressure (BVP) channel. For each of these 8 peripheral channels, we calculated the mean, variance, and entropy, resulting in a peripheral feature vector of 24 dimensions (8 channels × 3 features).

For the SEED-IV dataset, we extracted Differential Entropy (DE) features from the five preprocessed EEG frequency bands, also using an STFT with a 4-second non-overlapping Hanning window. For the 62 EEG channels, this process resulted in a final feature dimension of 62 × 5 = 310. The eye-tracking features, extracted from the SMI eye-tracking glasses, included both statistical and computed metrics. All 31 eye-tracking features used in this study are detailed in **Table 2**.

**Table 2:** Summary of extracted eye movement features.

Eye movement parameters	Extracted features
Pupil diameter (X and Y)	Mean, standard deviation, DE in four bands (0.2Hz, 0.2-0.4Hz, 0.4-0.6Hz, 0.6-1Hz)
Disperson (X and Y)	Mean, standard deviation
Fixation duration (ms)	Mean, standard deviation
Blink duration (ms)	Mean, standard deviation
Saccade	Mean and standard deviation of saccade duration (ms) and saccade amplitude (°)
Event statistics	Blink frequency fixation frequency fixation duration maximum fixation dispersion total fixation dispersion maximum saccade frequency saccade duration average saccade amplitude average saccade latency average

Differential Entropy (DE) is a feature that is frequently used in emotion recognition and has demonstrated excellent classification capability. DE is an extension of Shannon entropy to continuous variables, quantifying the total uncertainty of a continuous random variable’s probability distribution. It effectively reflects the frequency characteristics of EEG signals. An EEG signal within a short time interval can be approximated by a Gaussian distribution, and can thus be characterized by its Gaussian probability density function. The DE for an EEG signal that follows a Gaussian distribution is approximated as the logarithm of its power spectral density within a specific frequency band. The mathematical expression of this calculation is as follows by Equation 9:

(9) $$\begin{array}{ll} DE & =-\int_{a}^{b}f(x)\text{log }(f(x))dx\\ =-\int_{a}^{b}\frac{1}{\sqrt{2\pi \sigma^{2}}}\text{exp }\frac{{\left(x-\mu \right)}^{2}}{2\sigma^{2}}\text{log }(\frac{1}{\sqrt{2\pi \sigma^{2}}}\text{exp }\frac{{\left(x-\mu \right)}^{2}}{2\sigma^{2}})dx\\ =\frac{1}{2}\text{log }(2\pi e\sigma^{2}) \end{array}$$

## 3 Experiments and results

### 3.1 Experimental settings

**Figure 4:**
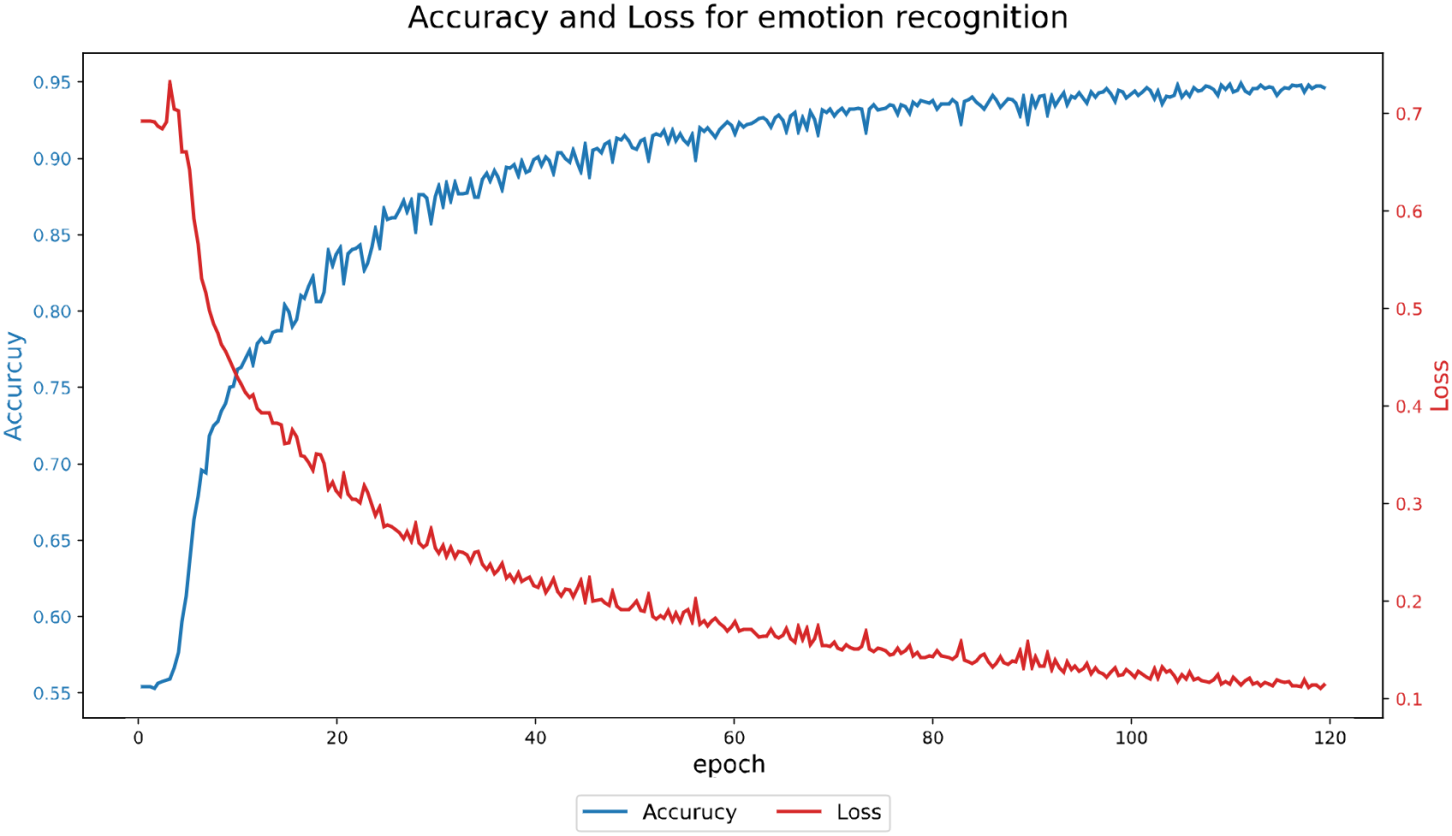
Accuracy and loss for EEG emotion recognition.

All experiments were conducted using the same hardware and software environment, data partitioning scheme, and hyperparameter settings to ensure consistency and fair comparison. The model was implemented on a hardware platform consisting of a Dell desktop computer equipped with an Intel Core i5-13400 @ 2.50GHz CPU and an Nvidia GeForce RTX 3060Ti GPU. The software environment was based on the Windows 10 operating system, with model implementation carried out in Python 3.9 using the PyTorch 1.10.1 deep learning framework. For the proposed model, the loss function was defined as the sum of cross-entropy loss and an L2 regularization term, which was minimized using the Adam optimizer. During the training process, the learning rate and batch size were set to 0.001 and 64, respectively. The **Figure 4** illustrates the training performance of emotion classification on the DEAP dataset.

For the DEAP dataset, three distinct classification experiments were performed: a binary classification task for valence (High Valence vs. Low Valence), a binary classification task for arousal (High Arousal vs. Low Arousal), and a four-class classification task based on the combined valence-arousal space (HAHV, HALV, LAHV, LALV). For the SEED-IV dataset, a four-class classification experiment was conducted to distinguish among happy, neutral, sad, and fear emotional states. Regarding the five-fold cross-validation on data from all subjects.

### 3.2 Results and comparison

**Figure 5:**
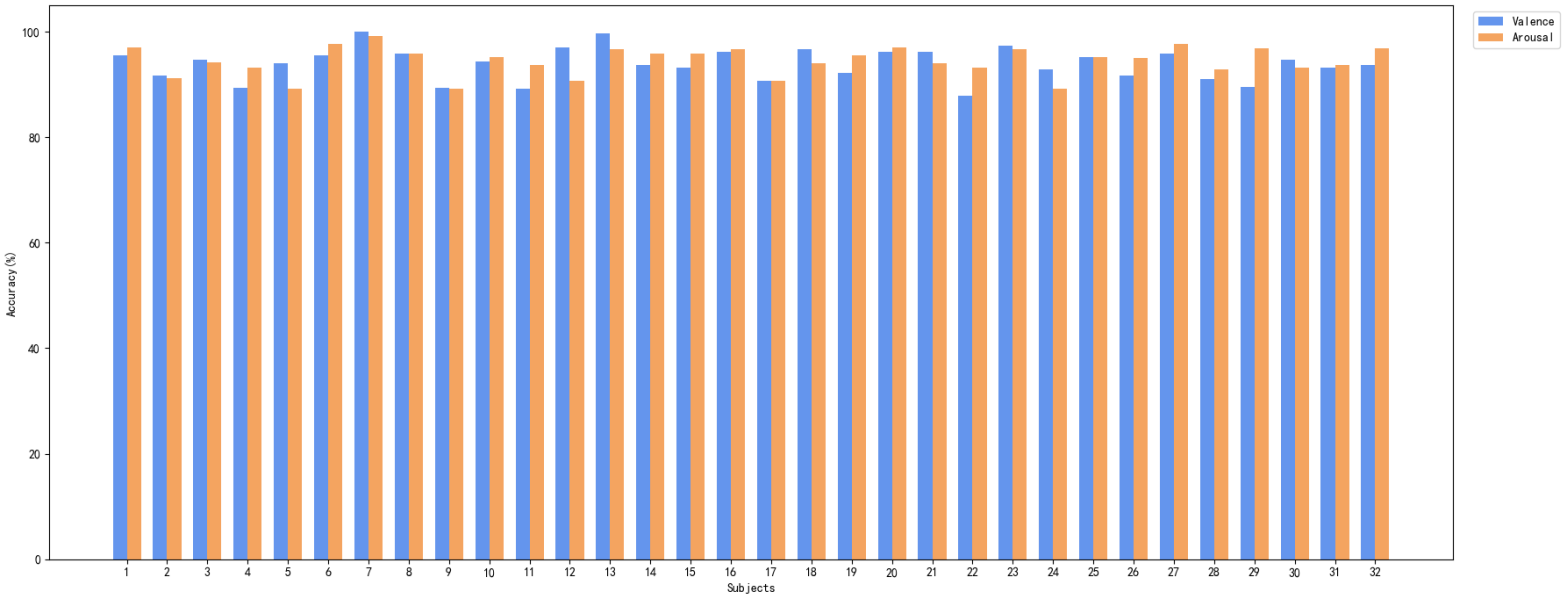
Comparison of recognition results for valence and arousal across all subjects in the DEAP dataset.

The experimental results on the DEAP dataset are presented in **Table 3**. The method proposed in this study achieved a mean accuracy of 94.88% on the valence dimension and 95.26% on the arousal dimension. The results indicate that the proposed model achieved the best performance among the compared methods. **Figure 5** presents the subject-dependent recognition accuracy results for the 32 participants in the DEAP dataset.

**Table 3:** Comparison of mean accuracies for valence and arousal classification on the DEAP dataset.

Method	Accuracy (%)	
	Valence	Arousal
BDAE[29]	85.20	80.50
DCCA[28]	85.62	84.33
HC-MFB[30]	90.46	93.22
MMResLSTM[31]	92.30	92.87
Ours	**94.88**	**95.26**

**Table 4** displays the results for the SEED-IV dataset. Four-class emotion recognition task (positive, neutral, and negative), the method proposed in this study achieved an accuracy of 89.32%. As shown in the table, this is the best result reported on the SEED-IV dataset to date. This demonstrates that the cross attention mechanism for multimodal signals proposed in this paper can fully leverage the informational richness and complementarity between different modalities. It not only improves the accuracy of emotion recognition but also better handles issues such as missing modalities and noise, thereby achieving accurate emotion classification from multimodal signals.

**Table 4:** Comparison of mean accuracies for emotion recognition on the SEED-IV dataset.

Method	Accuracy (%)
DCCA[28]	78.74
EmotionMeter[27]	85.11
MFFNN[32]	87.06
Ours	**89.12**

**Figure 6:**
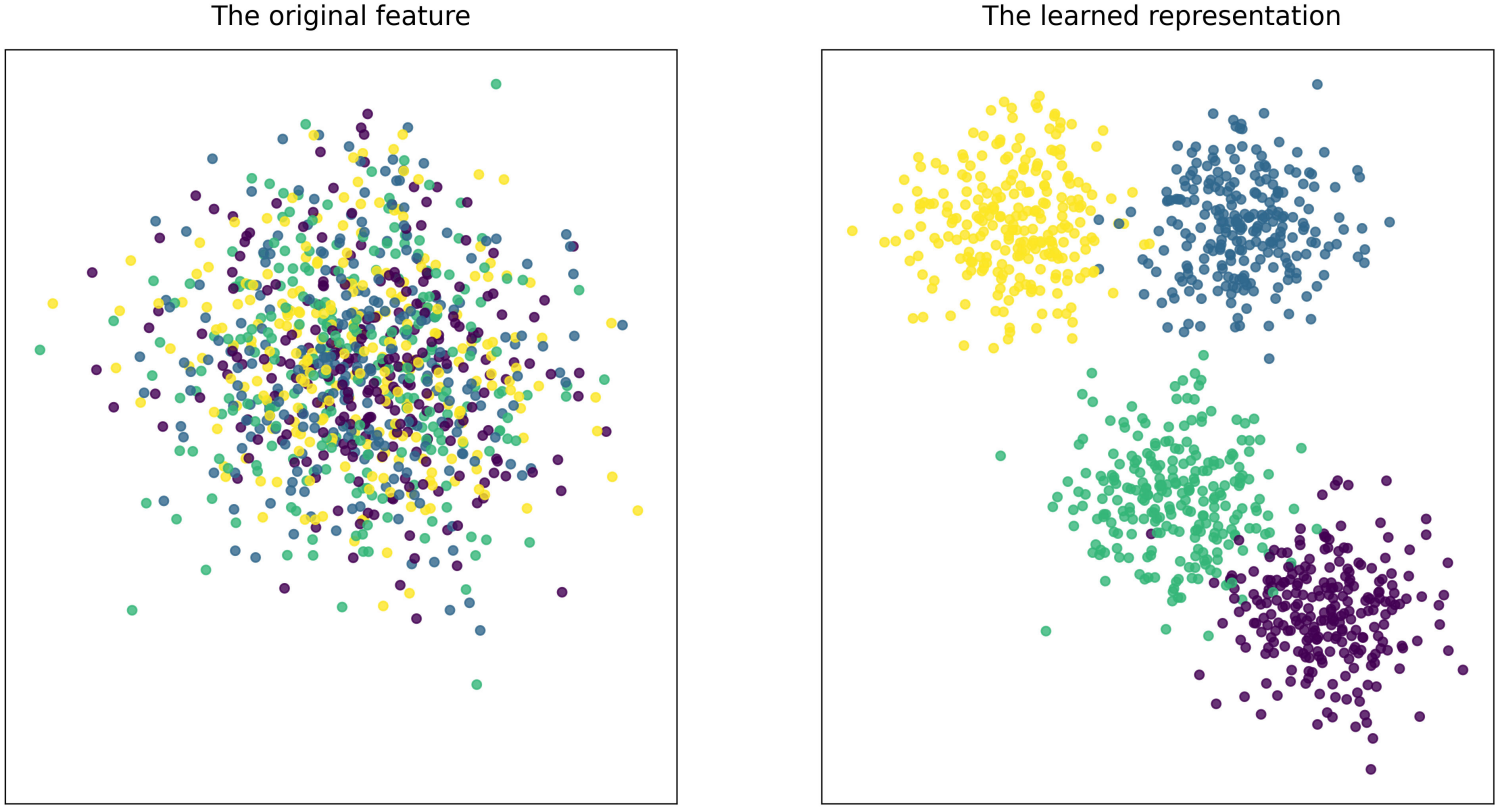
Feature visualization before and after training on the DEAP dataset.

To validate the efficacy of our proposed model in extracting high-level, abstract features, we performed a t-SNE visualization analysis. This analysis compared the two-dimensional distribution of Differential Entropy (DE) features from a subset of the dataset before and after being processed through our model, with the results depicted in **Figure 6**. As is evident in the figure, prior to feature extraction by our model, the samples corresponding to different emotional categories were severely intermingled and lacked clear separability. In stark contrast, following the deep feature extraction process, the sample distribution became highly structured. The data points formed distinct, well-defined clusters, leading to a significant reduction in sample confusion. This visual evidence powerfully substantiates our model’s capability to learn and extract potent, discriminative features that are strongly correlated with emotional states.

**Figure 7:**
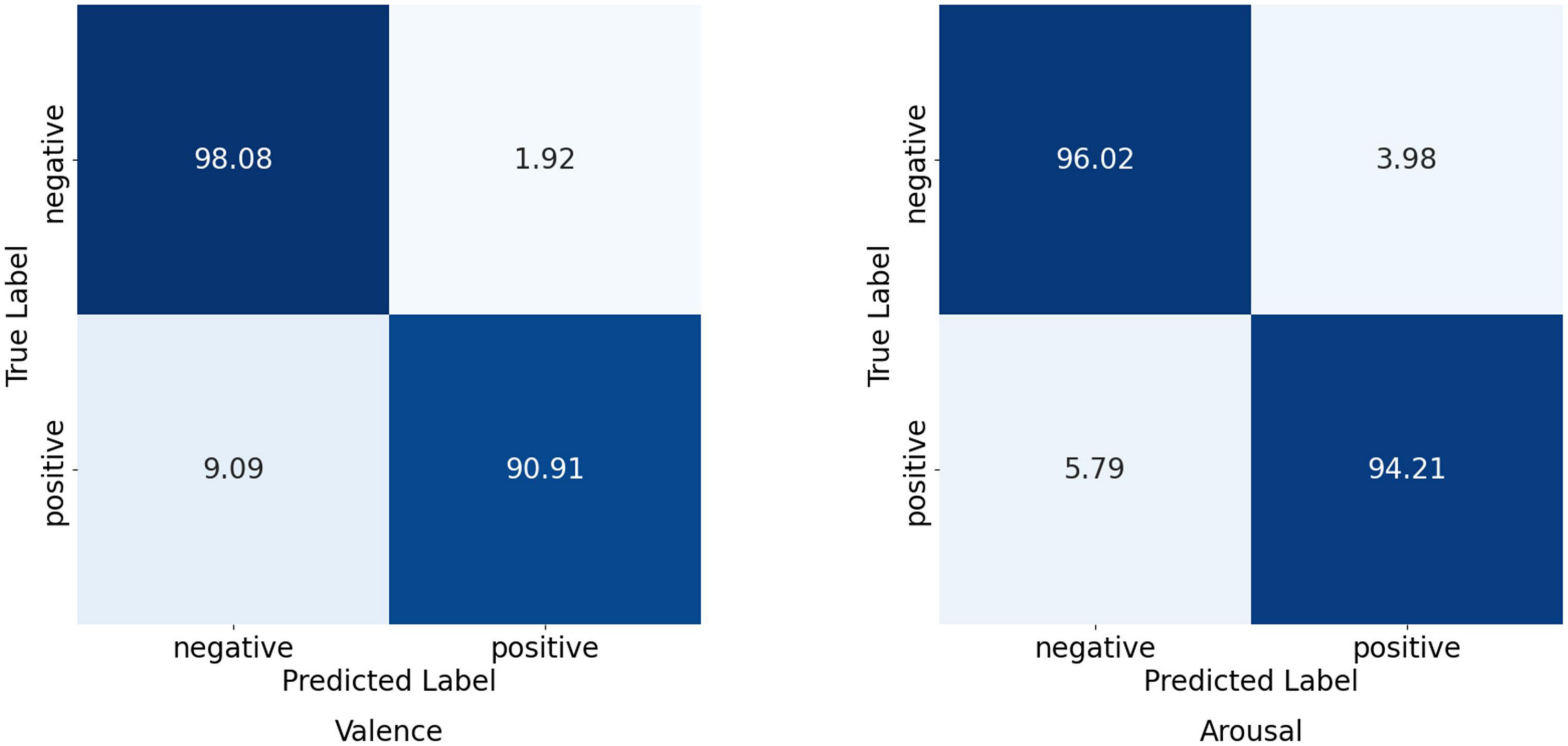
Confusion matrix of the classification results on the DEAP dataset.

To intuitively display the prediction performance for each emotion category across the different datasets, and to provide a clear comparison between the model’s predictions and the true labels for a deeper understanding of its performance, we computed confusion matrices for the results of our proposed model, as shown in **Figure 7**. In a confusion matrix, the sum of elements in each row represents the total number of samples for an actual class. The diagonal elements indicate the percentage of samples correctly classified for each emotion, while the off-diagonal elements represent the percentage of misclassified samples.

## 4 Discussion and conclusion

In this paper, we have proposed a novel framework for multimodal physiological emotion recognition that combines a dual-branch representation learning module with a multi-head cross attention mechanism. The experimental results on two benchmark multimodal physiological signal datasets, SEED-IV and DEAP, demonstrate that our model outperforms existing methods and achieves state-of-the-art results. On the DEAP dataset, we extracted features from the electroencephalography (EEG) and peripheral physiological signals, which were then fed into the dual-branch architecture to learn high-level representations for each modality. Subsequently, the multi-head cross attention mechanism was employed to fully leverage the richness and complementarity of the information, enabling accurate emotion recognition. The model’s efficacy was validated through binary classification experiments on the dimensions of valence and arousal, where it achieved the highest recognition performance. On the SEED-IV dataset, using EEG and eye-tracking signals as input, our model also attained the best recognition performance in a four-class classification task (happy, neutral, sad, and fear). The results across all subjects indicate that our proposed model can effectively process the multimodal data for every participant. Furthermore, the confusion matrices provided a clear comparison between the classification results and the ground-truth labels for each dataset. The aforementioned experimental results collectively prove that our proposed model can effectively process EEG, eye-tracking, and other peripheral physiological signals, successfully extracting salient features and fully utilizing both intra-modal and complementary information to achieve accurate emotion recognition.

The efficacy of this framework stems from its hierarchical and decoupled design philosophy. The dual-branch representation learning module first focuses on modeling intra-modal dynamics, providing high-quality feature sequences as input for the subsequent stage. On this foundation, the multi-head cross attention module then focuses on modeling inter-modal dynamic interactions. The effectiveness of the dual-branch architecture lies in its ability to ensure that the intrinsic characteristics of each modality are optimally and specifically extracted prior to fusion, thereby providing high-quality representations for subsequent computations. Unlike methods that output only static feature vectors, each branch in our model outputs a complete feature sequence that preserves temporal information, laying a solid foundation for capturing inter-modal correlations. The multi-head cross attention mechanism overcomes the limitations of traditional fusion methods, such as feature concatenation or averaging. It allows one modality’s representation sequence to adaptively query another, dynamically assigning attention weights to precisely capture the synergistic activities that occur during a genuine emotional response. Each attention head focuses on learning a specific type of cross-modal dependency, and by integrating these diverse and complementary correlation patterns, the model constructs a comprehensive and robust cross-modal representation, significantly enhancing its expressive power.

In summary, we have proposed a multimodal physiological emotion recognition framework that comprehensively learns from and extracts information across multiple modalities. We designed a dual-branch representation learning architecture to process EEG and peripheral signals in separate streams, providing ideal, decoupled inputs for feature fusion. We also introduced a cross attention mechanism tailored for multimodal signals, which leverages informational richness and complementarity to improve recognition accuracy while enhancing robustness to noise and missing data. The experimental results on the DEAP and SEED-IV datasets confirm that our proposed model exhibits superior performance compared to existing models in multimodal emotion classification tasks. The DEAP and SEED-IV datasets used in this study were both collected in controlled laboratory environments. The emotions induced via videos/music in such settings differ significantly from the complex, spontaneous emotions experienced in real life. Therefore, the model’s performance in real-world scenarios remains to be validated. We have added a discussion acknowledging that, as observed in our experimental results, the model’s performance fluctuates across different subjects. This indicates that achieving high-accuracy, subject-independent emotion recognition remains a significant challenge, and the model proposed in this paper has not yet fully overcome the problem of individual differences. Our findings demonstrate that the proposed model achieves effective extraction and fusion of multimodal physiological features. These results underscore the model’s significant potential in the field of emotion recognition and hold important implications for affective computing, healthcare, and human-computer interaction research. The insights gained from this work may provide a valuable reference for future studies in multimodal physiological signal analysis and affective Brain-Computer Interfaces (BCI). Despite the outstanding performance achieved in this study, there remains room for improvement. In our future work, we will explore alternative feature extraction methods and the fusion of an even wider range of signal modalities to further enhance the model’s adaptability and performance. While this paper validates the effectiveness of the multi-head cross-attention mechanism for fusing EEG and peripheral signals, we will not stop at merely using it as a “black box.” A key future direction is to conduct in-depth visualization and qualitative/quantitative analysis of the learned cross-attention weight matrices. This aims to explore, for specific emotional states, which dynamic patterns in the EEG signal the model has learned to strongly associate with which responses from the peripheral signals. This approach will enhance the model’s interpretability. Exploring Personalized Transfer Learning to Address Subject Variability. Our results reaffirm that “inter-subject variability” is a core challenge in this field. A future direction is to leverage the model proposed in this paper as a powerful general feature extractor and specifically investigate personalized transfer learning or domain adaptation techniques. For example, when encountering a new subject, we aim to see if we can use only a minimal amount of their calibration data to fine-tune specific layers of the model, enabling it to rapidly adapt to their unique physiological patterns.

## Data Availability

This study is an experimental analysis of publicly available datasets. The SEED-IV data can be found at this web page: https://bcmi.sjtu.edu.cn/home/seed/seed-iv.html and The DEAP data can be found at this web page: https://www.eecs.qmul.ac.uk/mmv/datasets/deap/.
